# Comparison of cardiac computed tomography recommendations in recent ESC vs. ACC/AHA guidelines

**DOI:** 10.1007/s10554-025-03375-0

**Published:** 2025-03-14

**Authors:** Nicolas Dayer, Nicola Ciocca, Panagiotis Antiochos, Henri Lu, Denise Auberson, David Meier, Pierre Monney, Christoph Gräni, David Rotzinger, Jonathon Leipsic, Georgios Tzimas

**Affiliations:** 1 Department of Cardiology, Lausanne University Hospital and University of Lausanne, 1011 Lausanne, Switzerland; 2 Department of Cardiology, Inselspital, Bern University Hospital, University of Bern, Bern, Switzerland; 3 Department of Diagnostic and Interventional Radiology, Lausanne University Hospital and University of Lausanne, Lausanne, Switzerland; 4 Department of Medicine and Radiology, University of British Columbia, Vancouver, BC Canada

**Keywords:** Cardiac CT, Clinical Guidelines, Coronary Disease, Valvular Disease, CCT Imaging

## Abstract

Cardiac computed tomography (CCT) continues to expand with increasing applications and technological advancements. Growing evidence on the clinical utility of CCT necessitates evaluating how this knowledge is incorporated into European Society of Cardiology (ESC) and American College of Cardiology (ACC)/American Heart Association (AHA) guidelines. We aimed to provide a comprehensive comparison of CCT indications between ESC and ACC/AHA guidelines to identify areas of consensus and divergence in the current landscape of CCT utilization. ESC and ACC/AHA guidelines were systematically reviewed for CCT recommendations. The class of recommendation (COR) and level of evidence (LOE) were compared using χ2 or Fisher exact tests. The latest ESC guidelines included 40 recommendations regarding CCT: 18 (45%) COR-I, 14 (35%) COR-IIa, 6 (15%) COR-IIb, and 2 (5%) COR-III. Two (5%) recommendation had LOE-A, 20 (50%) had LOE-B, and 18 (45%) had LOE-C. The latest ACC/AHA guidelines consisted of 54 recommendations: 18 (33.3%) COR-I, 28 (51.9%) COR-IIa, 6 (11.1%) COR-IIb, and 2 (3.7%) COR-III. Two recommendations were assigned LOE-A (3.7%), 30 (55.6%) were classified as LOE-B, and 22 (40.7%) as LOE-C. ACC/AHA guidelines had a significantly higher proportion of COR-IIa recommendations (*P* = 0.04) and similar proportions of COR-I and COR-IIb recommendations (*P* = 0.28; *P* = 0.76), compared to ESC guidelines. The proportion of LOE-B and LOE-C recommendations weren’t statistically different (*P* = 0.54; *P* = 0.84). ACC/AHA guidelines included more CCT recommendations with a higher COR and LOE than ESC guidelines. These findings highlight the need for continued research and consensus-building to establish standardized, evidence-based CCT recommendations in clinical practice.

**Supplementary Information:**

The online version contains supplementary material available at 10.1007/s10554-025-03375-0.

## Introduction

Cardiac computed tomography (CCT) has emerged as a first-line non-invasive imaging modality for diagnosing, evaluating the prognosis and guiding treatment in various cardiovascular diseases. As the field of CCT continues to evolve, guidelines from professional societies play a crucial role in providing recommendations for its appropriate use and integration into clinical decision-making. The European Society of Cardiology (ESC) and the American College of Cardiology/American Heart Association (ACC/AHA) independently develop guidelines for the diagnosis and management of cardiovascular diseases, serving as valuable resources for healthcare providers worldwide. However, they may differ in their recommendations due to variations in available evidence at the time, expert opinions, and regional considerations.

There is currently a mounting body of literature supporting the use of CCT in various cardiovascular conditions. The growing evidence on the clinical utility of CCT calls for a careful evaluation on how this evolving knowledge is incorporated into the ESC and ACC/AHA guidelines. Identifying and analyzing discrepancies in CCT indications are essential steps towards harmonizing guidelines and ensuring consistency in CCT utilization that may impact clinical decision-making and patient care.

Therefore, this study aims to provide a comprehensive comparison of CCT indications within the ESC and ACC/AHA guidelines, focusing specifically on cardiovascular diseases and conditions where CCT imaging is recommended. By conducting an evaluation of the similarities and differences, we aim to elucidate the current landscape of CCT utilization, identify areas of consensus and divergence, explore the underlying rationale, and assess the level of supporting evidence for the recommendations across both societies. This evaluation will help bridge the gap between research advancements and clinical practice, facilitating the appropriate integration of CCT as a powerful tool in the management of cardiovascular diseases.

## Methods

### Literature search and selection criteria

All the guidelines available on the ESC and ACC websites up to December 2024 were collected for analysis. Guidelines addressing conditions unrelated to the use of CCT (such as peripheral arterial disease, carotid and vertebral artery disease, pharmacology), and those which did not provide recommendations, were excluded. In case of overlap or replacement, the newer article was retained. The guidelines reviewed and included in the study are summarized in Supplemental Tables 1 and Supplemental Table 2, respectively.

To identify recommendations related to CCT, the guidelines were systematically screened using the following terms: “Cardiac computed tomography” or “Cardiac CT” or “cardiac computed tomography angiography” or “Coronary CT” or “CCT” or “CTA” or “CTCA“ or “CCTA” or “coronary artery calcium score” or “CAC” and if present, the relation to CCT was evaluated. The class of recommendation (COR) and level of evidence (LOE) for each recommendation were extracted. Recommendations that were not related to the use of CCT in cardiovascular diseases were excluded (e.g., non-cardiovascular CT imaging or vascular CT imaging for structures such as peripheral arteries or the aorta). The screening, eligibility, and extraction of recommendations was independently conducted by two authors (N.D., N.C.) and any discrepancy was resolved with the help of a third author (G.T.).

### Statistical analyses

Regarding cross-sectional comparisons, data were grouped into specific diagnostic subgroups (acute coronary syndrome [ACS], chronic coronary syndrome [CCS], heart failure [HF], arrhythmias, congenital heart disease [CHD], cardiomyopathies and pericardial disease, endocarditis, valvular heart disease [VHD], cardiovascular prevention, cardio-oncology, and sport cardiology) for direct comparisons between the latest versions of guidelines. COR and LOE were standardized across the ESC and AHA/ACC guidelines (Table 1). Finally, recommendations that were present in several guidelines were considered once, in the latest guideline document available.

**Table 1:**
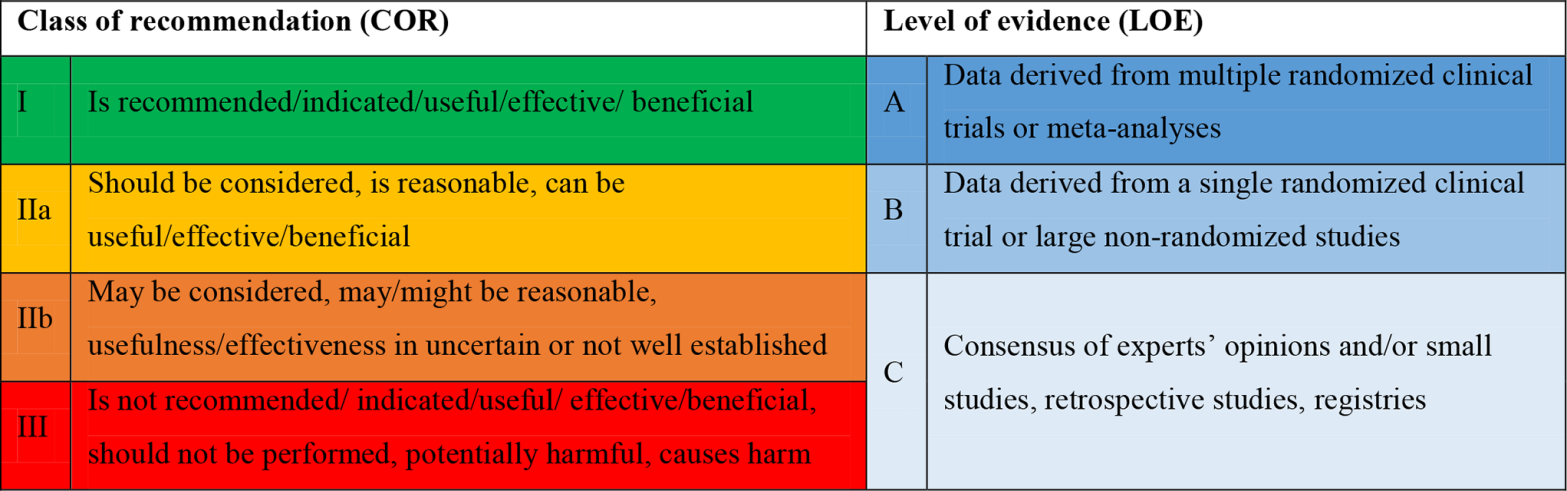
Definition of class of recommendation and level of evidence

Data were summarized using descriptive statistics, with frequencies and percentages for dichotomous variables. Comparison of categorical variables was assessed using the χ2 test, and if more than 20% of the expected cell counts were less than five, the Fisher exact test was used instead.

All p-values were two-sided, a value < 0.05 was used to define statistical significance. The analyses were performed using Stata Statistical Software, Release 17.0 (StataCorp. 2021. College Station, StataCorp LLC, TX, USA).

## Results

### Cross-sectional comparison of CCT recommendations in the latest ESC vs. ACC/AHA guidelines

**Fig. 1:**
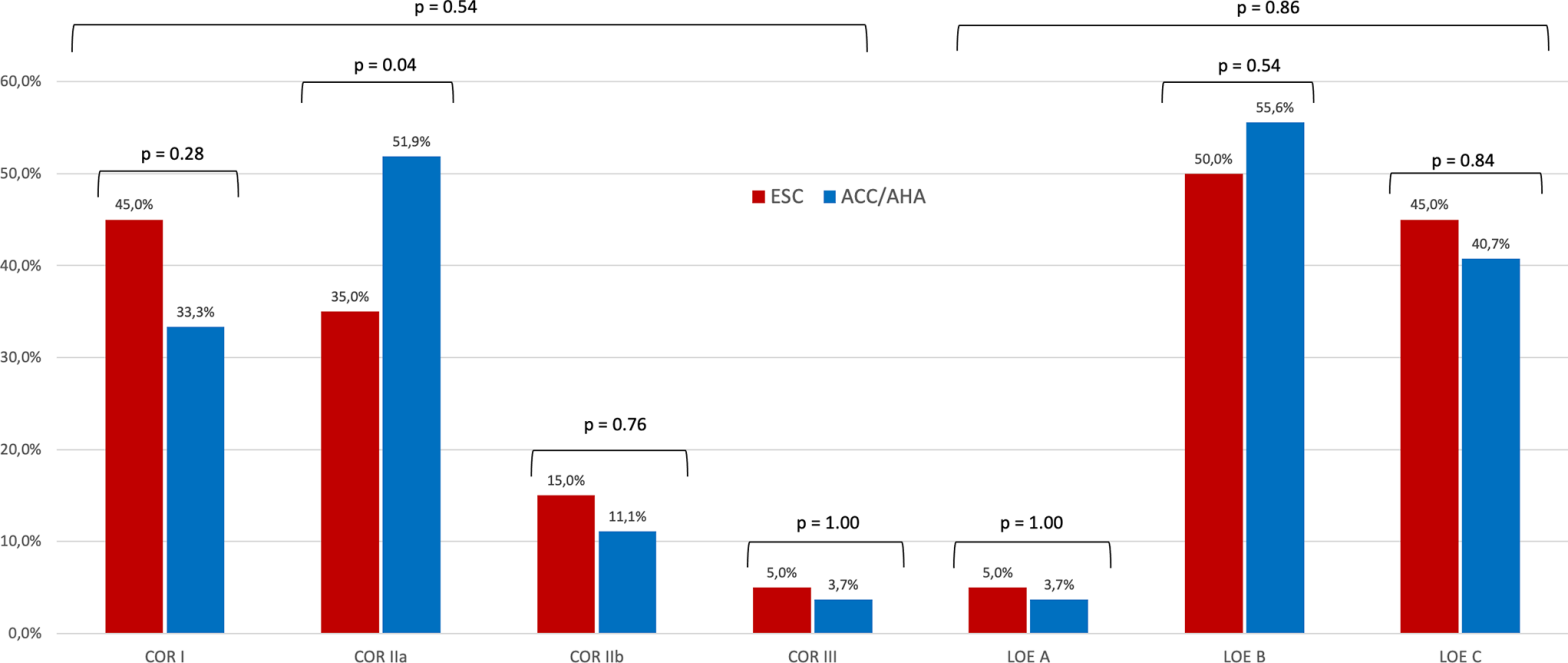
CCT recommendations in ESC vs. ACC/AHA up to 2024

Figure 1 shows the number of CCT recommendations in ESC vs. ACC/AHA guidelines, available in 2024, categorized by COR and LOE. The ESC guidelines included 40 recommendations: 18/40 (45%) COR I, 14/40 (35%) COR IIa, 6/40 (15%) COR IIb, and 2/40 (5%) COR III. Only two (5%) of the recommendations had LOE A, 20/40 (50%) LOE B and 18/40 (45%) LOE C. The latest ACC/AHA guidelines consisted of 54 recommendations: 18/54 (33.3%) COR I, 28/54 (51.9%) COR IIa, 6/54 (11.1%) COR IIb and 2/54 recommendation had COR III (3.7%). Two recommendations were assigned LOE A (2/54, 3.7%), 30/54 (55.6%) were classified as LOE B, and 22/54 (40.7%) as LOE C.

Overall, the distribution of COR was not statistically different between the guidelines (*P* = 0.54). The ACC/AHA guidelines had a statistically significant higher proportion of COR IIa recommendations compared with the ESC guidelines (51.9% vs. 35%; *P* = 0.04). Both guidelines showed a similar proportion of COR I and COR IIb recommendations (33.3% vs. 45%, *P* = 0.28; and 11.1% vs. 15%, *P* = 0.76, respectively).

The distribution of the LOE was not statistically different between the ESC and ACC/AHA guidelines (*P* = 0.86). The proportions of LOE-B and LOE-C recommendations were not statistically different (55.6% vs. 50%, *P* = 0.54; and 40.7% vs. 45%, *P* = 0.84, respectively). Both the ESC and ACC/AHA guidelines included a limited number of LOE A recommendations, comprising merely two in each case.

### Comparison of CCT recommendations by diagnostic subgroups

**Fig. 2:**
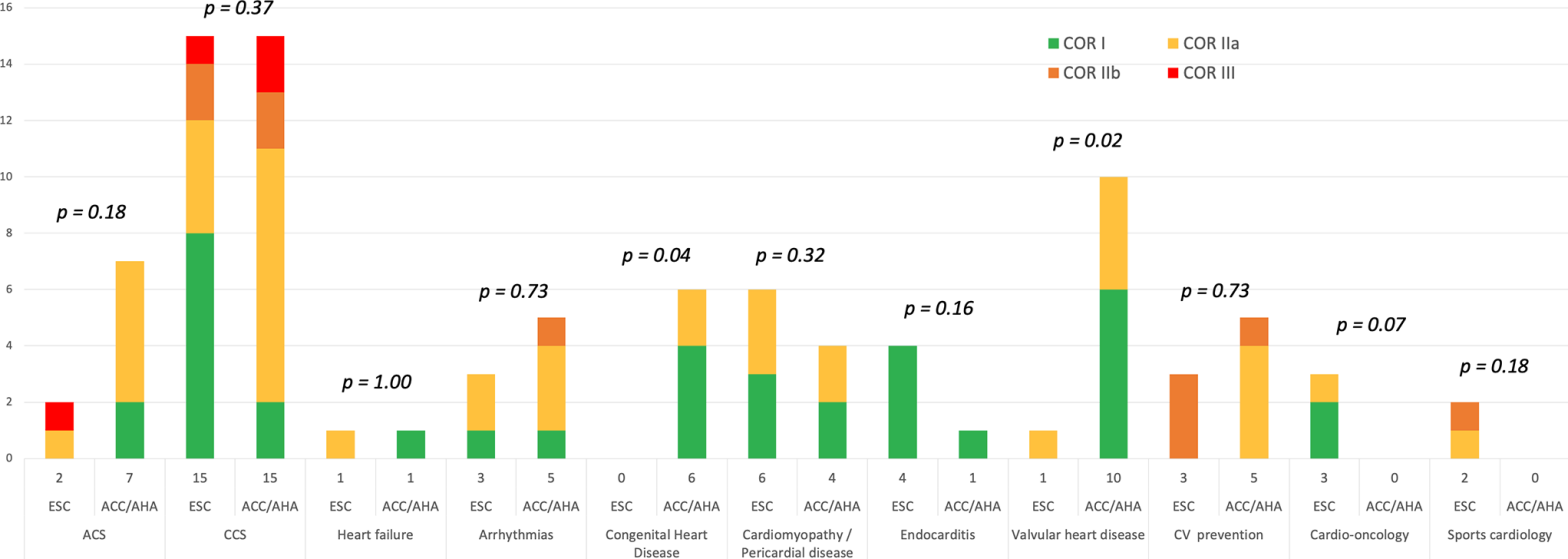
CCT recommendations in ESC vs. ACC/AHA by diagnosis groups

Figure 2 shows the number of recommendations issued by the ESC and the ACC/AHA, broken down by diagnostic subgroups.

#### Acute coronary syndrome (ACS)

For ACS, the ESC [1] and ACC/AHA guidelines [2] included 2 and 7 recommendations, respectively (Supplemental Table 3). There was a higher proportion of recommendations for CCT in ACS in the ACC/AHA guidelines, which did reach statistical significance (2 of 40, 5% vs. 7 of 54, 13%, *P* = 0.18). With regards to free text - without formal COR - in the guidelines, the ACC/AHA guidelines refer to several clinical situations in which CCT could be performed in patients with acute chest pain and intermediate to high risk of coronary artery disease. In addition, the use of Fractional Flow Reserve Computed Tomography (FFR-CT) is mentioned in ACC/AHA guidelines, but not in ESC guidelines. In contrast, the ESC guidelines only mention that CCT may be performed in patients with suspected ACS, low/negative high-sensitivity troponins, and normal ECG.

#### Chronic coronary syndrome (CCS)

Regarding CCS, the ESC [3–5] and ACC/AHA guidelines [2, 6, 7] included both 15 recommendations (Supplemental Table 4). The proportion of recommendation in this area was not statistically different (15 of 40, 37.5% vs. 15 of 54, 27.7%, *P* = 0.37). The recently published ESC guidelines included significantly higher COR I recommendations (8 vs. 2, *P* = 0.02). In the free text of the recommendations, we found a general consensus between ESC and AHA/ACC. Both societies agree that CCT is not recommended as a routine follow-up test for patients with established coronary artery disease.

#### Arrhythmias

In the context of arrhythmias, the ESC [8, 9] and ACC/AHA [10–12] guidelines included 3 and 5 recommendations, respectively (Supplemental Table 5). The proportion of recommendations in this field was not statistically different (3/40, 7.5% vs. 5/54, 9.3%; *P* = 0.73). When considering the free text of the guidelines, both the ESC and ACC/AHA agree on the role of CCT as an alternative imaging modality—alongside cardiac magnetic resonance and positron emission tomography-computed tomography—for the evaluation of structural heart disease.

#### Congenital heart disease

For congenital heart disease, the ACC/AHA guidelines [13] included 6 recommendations with a high proportion of COR I (66.7%), while the ESC guidelines [14] did not state any formal recommendations for the use of CCT (0/40, 0% vs. 6/54, 11.1%; *P* = 0.04) (Supplemental Table 6). In contrast, CCT is formulated in free text as an alternative diagnostic modality for specific indications such as assessment for coronary artery pathology, and detailed assessment of collaterals. The main areas of interest for the use of CCT following the ACC/AHA recommendations are anomalous coronary arteries, anomalous pulmonary venous connection, and aortic anomalies.

#### Cardiomyopathy & pericardial disease

Regarding cardiomyopathy and pericardial disease, the ESC [15], [16] and the ACC/AHA [2], [17] included 6 and 4 recommendations, respectively (Supplemental Table 7). The proportion of recommendations for CCT in cardiomyopathy and pericardial disease was not statistically different (15% vs. 7.4%, *P* = 0.32). In pericardial disease, both societies mention the use of CCT to determine the presence of pericardial thickening. In addition, both societies agree on the use of CCT for the further evaluation of cardiomyopathies or hypertrophic cardiomyopathy as an alternative modality when cardiac magnetic resonance imaging is contraindicated or not available.

#### Valvular heart disease

Regarding VHD, the ESC [18] and the ACC/AHA [19] guidelines included 1 and 10 recommendations, respectively (Supplemental Table 8). The proportion of recommendations for the use of CCT in VHD was higher in the ACC/AHA group and statistically significant (2.5% vs. 18.5%, *P* = 0.02). The ESC guidelines did not include any COR I, while the ACC/AHA included 6 COR I. Both ESC and ACC/AHA support the use of CCT in patients with low-flow, low-gradient aortic stenosis to further define severity. In addition, the ACC/AHA include recommendations for the use of CCT in suspected mechanical/bioprosthetic valve thrombosis/stenosis and to rule out leaflet thrombosis.

#### Cardiovascular prevention and sports cardiology

Regarding cardiovascular prevention, the ESC [20], [21], [22] and the ACC/AHA [23], [24] guidelines included 3 and 5 recommendations, respectively (Supplemental Table 9). The proportion of recommendations was not statistically different (7.5% vs. 9.3%, *P* = 0.73). Both guidelines agree on the use of CCT to calculate coronary artery calcium (CAC) score in patients at intermediate cardiovascular risk to guide further therapeutic decisions. In sports cardiology, the ESC guidelines [25] included 2 recommendations for the use for CCTA. The ACC/AHA guidelines didn’t state any recommendation (Supplementary Table 10).

#### Endocarditis and cardio-oncology

In the context of endocarditis, the ESC guidelines [26] included 4 recommendations and the ACC/AHA guidelines [19] one (Supplemental Table 11). The proportion of recommendations was not statistically different between the ESC and ACC/AHA (10% vs. 1.8%, *P* = 0.16). CCT is recommended in patients with possible native and prosthetic valve endocarditis to detect valvular lesions as well as paravalvular or periprosthetic complications and confirm the diagnosis of infective endocarditis. Regarding cardio-oncology, the ESC guidelines [27] included 3 recommendations with a high proportion of COR I (2/3, 66.6%) (Supplemental Table 12). The ACC/AHA guidelines did not state any recommendation for the use of CCT in this field. Although not statistically significant, these are two of the only areas where ESC had more recommendations than AHA/ACC.

#### Heart failure

Regarding heart failure, both the ESC [28] and ACC/AHA [29] guidelines included one recommendation each (Supplemental Table 13). The ESC guidelines emphasize the role of CCT in patients with heart failure and a low to intermediate pretest probability of coronary artery disease to rule out coronary artery stenosis. In contrast, the ACC/AHA guidelines mention CCT only as an alternative imaging modality to estimate left ventricular ejection fraction when transthoracic echocardiography is inconclusive.

## Discussion

**Fig. 3:**
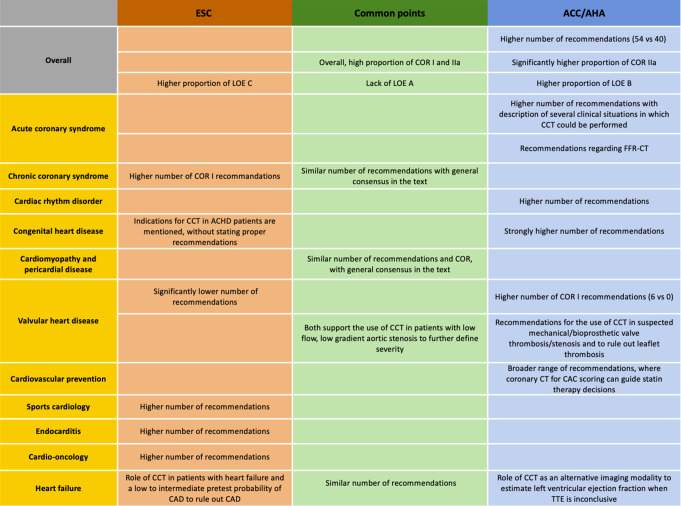
Central figure summarizing the main points of the article

Our analysis of the ACC/AHA and ESC guidelines reveals significant differences in their recommendations for the use of CCT. This study highlighted several points of consensus and divergence (Fig. 3).

The ACC/AHA guidelines provide a broader range of recommendations for the use of CCT than the ESC guidelines, highlighting the ACC/AHA’s broader endorsement of CCT in clinical practice. The ACC/AHA assigns a higher COR to CCT-related guidelines, with a significantly higher proportion of COR IIa recommendations and a lower proportion of COR I. Similarly, the ACC/AHA appears to attribute a higher level of evidence (LOE) to its recommendations. This suggests that while both societies recognize the strong indications for CCT, the ACC/AHA experts demonstrate greater confidence in its applicability.

An interesting observation relates to the low number of LOE A recommendations in both guidelines (two in the ESC and in the ACC/AHA, respectively) despite the publication of high-level evidence in the literature for CCT. For instance, despite the increase in transcatheter aortic valve replacement procedures, neither the ESC nor ACC/AHA guidelines include recommendations for the use of CCT in the pre-diagnostic work-up for transcatheter aortic valve replacement. Currently, guidance on this matter is provided by an expert consensus from the Society of Cardiovascular Computed Tomography [30]. Likewise, despite the potential benefits, there are no current recommendations for the use of CT in the pre-procedural workup for transcatheter mitral valve replacement. CT can be crucial in identifying patients with an increased anatomical risk for small neo-left ventricular outflow tract dimensions and potential obstruction of the outflow tract, which can result in adverse clinical outcomes [31]. This cautious approach indicates that international societies are awaiting more comprehensive and robust clinical evidence before elevating the strength of their recommendations, highlighting the need for large, multicenter randomized trials to address existing gaps in knowledge and provide the necessary evidence to refine clinical guidance for CCT across all domains.

The ACC/AHA guidelines provide more comprehensive recommendations for the use of CCT in ACS. In contrast, the ESC recommendations are more restrictive in this area, limiting the use of CCT to patients with suspected ACS, non-elevated (or uncertain) high-sensitivity cardiac troponin, no ECG changes, and no recurrence of pain (COR IIa, LOE A). According to the ESC guidelines, routine, early CCT in patients with suspected ACS is not recommended. In contrast, the ACC/AHA guidelines include several clinical situations to guide physicians in their decision-making for patients with suspected ACS. They recommend CCT for the exclusion of atherosclerotic plaque and obstructive coronary artery disease in intermediate-risk patients with acute chest pain and no known coronary artery disease after a negative or inconclusive evaluation for ACS (COR I, LOE A). In addition, CCT should be considered for intermediate-risk patients with acute chest pain and no known coronary artery disease for diagnosing obstructive coronary disease if previous (< 1 year) mildly abnormal or inconclusive stress test results are present. Lastly, they recommend using CCT in patients with prior coronary artery bypass grafting surgery presenting with acute chest pain to evaluate graft stenosis or occlusion.

Although the current ESC recommendations on chronic coronary syndromes (CCS) do not recommend CCT as a routine follow-up test for patients with established coronary artery disease, the ACC/AHA guidelines include different scenarios for its use in patients presenting with stable chest pain and known obstructive coronary artery disease, regardless of previous revascularization (stent diameter ≥ 3 mm) or prior coronary artery bypass grafting. Additionally, they state that in symptomatic patients with known non-obstructive coronary artery disease presenting with stable chest pain, CCT should be considered for determining atherosclerotic plaque burden and progression to obstructive coronary artery disease, thereby guiding therapeutic decision-making. Regarding strong recommendations (COR I, LOE A), the ACC/AHA guidelines recommend using CCT for diagnosis of CAD, risk stratification and guiding treatment decisions in intermediate high-risk patients with stable chest pain and no known CAD. In contrast, the ESC guidelines take a different stance, focusing on the utility of CCT primarily for diagnosing obstructive CAD and estimating the risk of major adverse cardiac events in patients with suspected CCS and a low to moderate (> 5–50%) pre-test likelihood of obstructive CAD. This divergence reflects differing perspectives on the optimal application of CCT across varying risk profiles. Although several multinational registries have examined the utility of FFR-CT with regard to guiding clinical decision-making and the safety of deferring coronary revascularization in patients with a negative FFR-CT, FFR-CT is not yet recommended in the ESC guidelines [32]. On the opposite, ACC/AHA guidelines state that FFR-CT should be considered useful for the diagnosis of vessel-specific ischemia and to guide decision-making regarding the coronary revascularization in intermediate-high risk patients with stable chest pain and known coronary stenosis of 40–90% in a proximal or middle coronary segment on CCT.

Similarly, the ACC/AHA guidelines provide a considerably higher number of recommendations for CCT in congenital heart disease, while the ESC guidelines make no recommendations in this area. CCT is considered an alternative imaging modality in this area, where patients should be protected from ionizing radiation due to repeated imaging. However, CCT is considered superior to cardiac magnetic resonance in areas such as coronary anomalies and coronary artery disease (e.g., intramural course, slit-like course, myocardial bridging) due to the higher spatial resolution, which is key in small vessels. A difference in how experts choose to convey key messages could explain the absence of recommendations for CCT in the ESC guidelines for adult congenital heart disease.

The use of CT to calculate CAC score and tailor statin therapy in primary prevention is well-supported by the guidelines, reinforcing its role in preventive cardiology. Although the ESC guidelines recommend using CAC to adjust the cardiovascular risk classification of asymptomatic individuals at low or moderate risk, there is a difference in approach in North America. In the ACC/AHA, the CAC score is used to make treatment decisions regarding statin use in intermediate-risk patients. The presence and severity of CAC may help stratify patients who are most likely to benefit from statins [33], [34] Conversely, if CAC is zero, discussions about withholding statin therapy can be considered for intermediate-risk patients [35], [36] The common treatment threshold for considering or initiating statin therapy is a CAC score greater than 100 [37]. 

## Limitations

Despite our comprehensive review, some limitations need to be acknowledged. Firstly, our study focused solely on the ESC and ACC/AHA guidelines, and thus, our findings may not reflect the global landscape of CCT utilization. It is possible that other regional or specialty-specific guidelines may have different perspectives and recommendations regarding CCT use. Secondly, the guideline documents themselves are subject to periodic updates, and our analysis was based on the most recent versions available at the time of our study. Future guidelines revisions may alter the number and content of CCT recommendations. Thirdly, although we compared the number, COR, and LOE of CCT recommendations, our study did not delve into the specific clinical contexts and indications for CCT use in each guideline, as they were sometimes only described in the text. Fourthly, we have subjectively evaluated the guidelines and excluded non-cardiovascular CT imaging or vascular CT imaging. Additionally, we acknowledge that differences in the total number of recommendations between ESC and ACC/AHA guidelines could influence the relative proportions of CCT recommendations, and this should be considered when interpreting our findings. Lastly, we did not compare the number or strength of recommendations for other imaging modalities such as echocardiography, cardiac magnetic resonance, positron emission tomography, or single-photon emission computed tomography. Future studies could explore this comparison to provide a more comprehensive evaluation of the relative strength of recommendations for CCT in the context of multimodality imaging.

## Conclusion

In conclusion, the comparative analysis of ACC/AHA and ESC guidelines on CCT underscores significant divergences in their recommendations. The ACC/AHA guidelines encompassed a greater number of recommendations for CCT utilization and attributed a higher COR and better LOE to their CCT recommendations than ESC guidelines. These findings and the differences found in the analysis of diagnostic subgroups, emphasize the need for continued research and consensus-building efforts to establish standardized approaches and evidence-based recommendations for CCT use in clinical practice. Future guideline updates should consider incorporating emerging evidence and refining the clinical guidance for CCT, considering the variations in available evidence and regional perspectives.

## Electronic supplementary material

Below is the link to the electronic supplementary material.

Supplementary Material 1

## Data Availability

No datasets were generated or analysed during the current study.

## Abbreviations

ACC: American College of Cardiology
ACS: Acute Coronary Syndrome
AHA: American Heart Association
CAC: Coronary Artery Calcium
CAD: Coronary Artery Disease
CCS: Chronic Coronary Syndrome
CCT: Cardiac Computed Tomography
CHD: Congenital Heart Disease
COR: Class of Recommendation
HF: Heart Failure
LOE: Level of Recommendation
ESC: European Society of Cardiology
VHD: Valvular Heart Disease
