# Supplementary material for: Comparison of cardiac computed tomography recommendations in recent ESC vs. ACC/AHA guidelines
Source: Int J Cardiovasc Imaging. 2025 Mar 14;41(5):933–41. doi: 10.1007/s10554-025-03375-0 (PMC12075283; doi:10.1007/s10554-025-03375-0)
Supplement: Supplementary file 1 — Supplementary Material 1 [file 10554_2025_3375_MOESM1_ESM.docx]

## **Supplemental material**

**Supplemental Table 1**: ESC and ACC/AHA guidelines reviewed for the article.

| ESC | ACC/AHA |
| --- | --- |
| 2024 High blood pressure and hypertension ^1^ | 2024 Primary prevention of stroke ^2^ |
| 2024 Chronic coronary syndrome ^3^ | 2024 Perioperative cardiovascular management for noncardiac surgery ^4^ |
| 2024 Atrial fibrillation ^5^ | 2024 Hypertrophic cardiomyopathy ^6^ |
| 2024 Peripheral arterial and aortic disease ^7^ | 2023 Atrial fibrillation ^5^ |
| 2023 Focused update acute and chronic heart failure ^8^ | 2023 Chronic coronary syndrome ^9^ |
| 2023 Acute coronary syndrome ^10^ | 2022 Aortic disease ^11^ |
| 2023 Cardiomyopathies ^12^ | 2022 Heart failure ^13^ |
| 2023 Cardiovascular disease and diabetes ^14^ | 2021 Coronary artery revascularization ^15^ |
| 2023 Endocarditis ^16^ | 2021 Chest pain ^17^ |
| 2022 Cardio-oncology ^18^ | 2020 Valvular heart disease ^19^ |
| 2022 Ventricular arrhythmias and prevention of sudden cardiac death ^20^ | 2019 Innovations, modifications, and evolution of ACC/AHA Clinical practice guidelines ^21^ |
| 2022 Non-cardiac surgery: cardiovascular assessment and management ^22^ | 2019 Primary prevention ^23^ |
| 2022 Pulmonary hypertension ^24^ | 2018 Blood cholesterol ^25^ |
| 2021 Cardiovascular disease prevention in clinical practice ^26^ | 2018 Bradycardia and cardiac conduction delay ^27^ |
| 2021 Cardiac pacing & CRT ^28^ | 2018 Adults with congenital heart disease ^29^ |
| 2021 Valvular heart disease ^30^ | 2017 High blood pressure in adults ^31^ |
| 2021 Heart failure ^32^ | 2017 Ventricular arrhythmias and prevention of sudden cardiac death ^33^ |
| 2020 Sports cardiology and exercise in patients with cardiovascular disease ^34^ | 2017 Syncope ^35^ |
| 2020 Management of adult congenital heart disease ^36^ | 2017 Clinical practice guideline implementation strategies ^37^ |
| 2019 Supraventricular tachycardia ^38^ | 2016 DAPT in patients with coronary artery disease (Focused Update) ^39^ |
| 2019 Dyslipidemias ^40^ | 2015 Clinical practice guideline recommendation classification System ^41^ |
| 2019 Acute pulmonary embolism ^42^ | 2015 Supraventricular tachycardia ^43^ |
| 2018 ESC/EACTS myocardial revascularization ^44^ | 2014 Non-ST-elevation ACS ^45^ |
| 2018 Cardiovascular disease during pregnancy ^46^ | 2014 Clinical practice guidelines in patients with cardiovascular disease and comorbid conditions ^47^ |
| 2018 Syncope ^48^ | 2014 Clinical practice guidelines, the evolution and future: A 30-year journey ^49^ |
| 2017 Focused update on DAPT ^50^ | 2012 ST-elevation myocardial Infarction ^51^ |
| 2015 Pericardial disease ^52^ |  |
| 2007 The Role of endomyocardial Biopsy in the management of cardiovascular disease ^53^ |  |

**Supplemental Table 2**: ESC and ACC/AHA guidelines included in the article.

| ESC | ACC/AHA |
| --- | --- |
| 2024 High blood pressure and hypertension ^1^ | 2024 Perioperative cardiovascular management for noncardiac surgery ^4^ |
| 2024 Chronic coronary syndrome ^3^ | 2024 Hypertrophic cardiomyopathy ^6^ |
| 2024 Atrial fibrillation ^5^ | 2023 Chronic coronary syndrome ^9^ |
| 2023 Acute coronary syndrome ^10^ | 2022 Heart failure ^13^ |
| 2023 Cardiomyopathies ^12^ | 2021 Chest pain ^17^ |
| 2023 Endocarditis ^16^ | 2020 Valvular heart disease ^19^ |
| 2022 Cardio-oncology ^18^ | 2019 Primary prevention ^23^ |
| 2022 Non-cardiac surgery: cardiovascular assessment and management ^22^ | 2018 Blood cholesterol ^25^ |
| 2021 Cardiovascular disease prevention in clinical practice ^26^ | 2018 Bradycardia and cardiac conduction delay ^27^ |
| 2021 Cardiac pacing & CRT ^28^ | 2018 Adults with congenital heart disease ^29^ |
| 2021 Valvular heart disease ^30^ | 2017 Ventricular arrhythmias and prevention of sudden cardiac death ^33^ |
| 2021 Heart failure ^32^ | 2017 Syncope ^35^ |
| 2020 Sports cardiology and exercise in patients with cardiovascular disease ^34^ |  |
| 2020 Management of adult congenital heart disease ^36^ |  |
| 2019 Dyslipidemias ^40^ |  |
| 2015 Pericardial disease ^52^ |  |

**Supplemental Table 3**: ESC and ACC/AHA recommendations for Acute Coronary Syndrome (ACS) in 2024

| **ESC** | **COR / LOE** | | **ACC / AHA** |
| --- | --- | --- | --- |
| **Recommendations for imaging for patients with suspected NSTE-ACS** |  |  | **Intermediate-Risk Patients with Acute Chest Pain and No Known CAD** |
| In patients with suspected ACS, non-elevated (or uncertain) hs-cTn, no ECG changes and no recurrence of pain, incorporating CCTA or a non-invasive stress imaging test as part of the initial workup should be considered. | IIa / A | 1 / A | For intermediate-risk patients with acute chest pain and no known CAD eligible for diagnostic testing after a negative or inconclusive evaluation for ACS, CCTA is useful for exclusion of atherosclerotic plaque and obstructive CAD |
| Routine, early CCTA in patients with suspected ACS is not recommended. | III / B | 2a / C | For intermediate-risk patients with acute chest pain with evidence of previous mildly abnormal stress test results (<1year), CCTA is reasonable for diagnosing obstructive CAD |
|  |  | 2a / C | For intermediate-risk patients with acute chest pain and no known CAD, as well as an inconclusive prior stress test, CCTA can be useful for excluding the presence of atherosclerotic plaque and obstructive CAD. |
|  |  | 2a / B | For intermediate-risk patients with acute chest pain and no known CAD, with a coronary artery stenosis of 40% to 90% in a proximal or middle coronary artery on CCTA, FFR-CT can be useful for the diagnosis of vessel-specific ischemia and to guide decision-making regarding the use of coronary revascularization |
|  |  |  | **Intermediate-Risk Patients with Acute Chest Pain and Known CAD** |
|  |  | 2a / B | For intermediate-risk patients with acute chest pain and known non obstructive CAD, CCTA can be useful to determine progression of atherosclerotic plaque and obstructive CAD |
|  |  | 2a / B | For intermediate-risk patients with acute chest pain and coronary artery stenosis of 40% to 90% in a proximal or middle segment on CCTA, FFR-CT is reasonable for diagnosis of vessel-specific ischemia and to guide decision-making regarding the use of coronary revascularization |
|  |  |  | **Chest Pain in prior CABG** |
|  |  | 1 / C | In patients with prior CABG surgery presenting with acute chest pain who do not have ACS, performing stress imaging is effective to evaluate for myocardial ischemia or CCTA for graft stenosis or occlusion |

**Supplemental Table 4**: ESC and ACC/AHA recommendations for Chronic Coronary Syndrome (CCS) in 2024

| **ESC** | **COR / LOE** | | **ACC / AHA** |
| --- | --- | --- | --- |
| **CV assessment before non-cardiac surgery** |  |  | **CV assessment before non-cardiac surgery** |
| CCTA should be considered to rule out CAD in patients with suspected CCS or biomarker-negative NSTE-ACS in case of low-to-intermediate clinical likelihood of CAD, or in patients unsuitable for non-invasive functional testing undergoing non-urgent, intermediate-, and high-risk NCS. | IIa / C | 2b / B | For patients undergoing elevated-risk surgery with poor or unknown functional capacity, and elevated risk for perioperative cardiovascular events based on a validated risk tool, CCTA for the detection of high-risk coronary anatomy may be considered. |
|  |  | 3 / B | In patients who are at low risk for perioperative cardiovascular events, have adequate functional capacity with stable symptoms, or who are undergoing low-risk procedures, routine CCTA before NCS is not recommended due to lack of benefit. |
| **Diagnostic** |  |  | **Diagnostic** |
| In individuals with suspected CCS and low or moderate (>5%–50%) pre-test likelihood of obstructive CAD, CCTA is recommended to diagnose obstructive CAD and to estimate the risk of MACE. | I / A | 2a / B | In patients with CCD and a change in symptoms or functional capacity that persists despite GDMT, and who have had previous coronary revascularization, coronary CT angiography (CCTA) is reasonable to evaluate bypass graft or stent patency (for stents ≥ 3 mm) |
| CCTA is recommended in individuals with low or moderate (>5%–50%) pre-test likelihood of obstructive CAD to refine diagnosis if another non-invasive test is non-diagnostic. | 1 / B |  | **Low-Risk Patients with Stable Chest Pain and No Known CAD** |
| CCTA is not recommended in patients with severe renal failure (eGFR <30 mL/min/1.73 m2), decompensated heart failure, extensive coronary calcification, fast irregular heart rate, severe obesity, inability to cooperate with breath-hold commands, or any other conditions that can make obtaining good imaging quality unlikely. | III / C | 2a / B | For patients with stable chest pain and no known CAD categorized as low risk, CAC testing is reasonable as a first-line test for excluding calcified plaque and identifying patients with a low likelihood of obstructive CAD |
| In symptomatic patients in whom the pre-test likelihood of obstructive CAD by clinical assessment is >5%, CCTA or non-invasive functional imaging for myocardial ischaemia is recommended as the initial diagnostic test | I / B |  | **Intermediate-High Risk Patients With Stable Chest Pain and No Known CAD** |
| To rule out obstructive CAD in individuals with low or moderate (>5%–50%) pre-test likelihood, CCTA is recommended as the preferred diagnostic modality. | I / B | 1 / A | For intermediate-high risk patients with stable chest pain and no known CAD, CCTA is effective for diagnosis of CAD, for risk stratification, and for guiding treatment decisions |
| CCTA is recommended in individuals with low or moderate (>5%–50%) pre-test likelihood of obstructive CAD if functional imaging for myocardial ischaemia is not diagnostic. | I / B | 2a / B | For intermediate-high risk patients with stable chest pain and known coronary stenosis of 40% to 90% in a proximal or middle coronary segment on CCTA, FFR-CT can be useful for diagnosis of vessel-specific ischemia and to guide decision-making regarding the use of coronary revascularization |
| In patients with a known intermediate coronary artery stenosis in a proximal or mid coronary segment on CCTA, CT-based FFR may be considered. | IIb / B | 2a / B | For intermediate-high risk patients with stable chest pain after an inconclusive or abnormal exercise ECG or stress imaging study, CCTA is reasonable |
| **Likelihood of obstructive atherosclerotic coronary artery disease in the initial diagnostic management of individuals with suspected chronic coronary syndrome** |  | 2a / B | For intermediate-high risk patients with stable chest pain and no known CAD undergoing stress testing, the addition of CAC testing can be useful |
| In individuals with a *low* (>5%–15%) pre-test likelihood of obstructive CAD, CACS should be considered to reclassify subjects and to identify more individuals with very low (≤5%) CACS-weighted clinical likelihood. | IIa / B | 2a / B | For intermediate-high risk patients with stable chest pain after inconclusive CCTA, stress imaging is reasonable |
| **Definition of high risk of adverse events** |  | 2b / C | For intermediate-high risk patients with stable chest pain after a negative stress test but with high clinical suspicion of CAD, CCTA or ICA may be reasonable |
| The use of one or more of the following test results is recommended to identify individuals at high risk of adverse events: [...][CCTA:   - left main disease with ≥50% stenosis, - two-vessel disease with ≥70% stenosis, including the proximal LAD or one-vessel disease of the proximal LAD with ≥70% stenosis and FFR-CT ≤0.8 | I / B |  | **Patients with Obstructive CAD Who Present With Stable Chest Pain** |
| **Management of chronic coronary syndrome patients with chronic heart failure** |  | 1 / B | For symptomatic patients with obstructive CAD who have stable chest pain with CCTA-defined >50% stenosis in the left main coronary artery, obstructive CAD with FFR with CT <0.80, or severe stenosis (>70%) in all 3 main vessels, ICA is effective for guiding therapeutic decision-making |
| - In HF patients with LVEF >35% and suspected CCS with low or moderate (>5%–50%) pre-test likelihood of obstructive CAD, CCTA or functional imaging is recommended. | I / C | 2a / B | For patients who have stable chest pain with previous coronary revascularization, CCTA is reasonable to evaluate bypass graft or stent patency (for stents>3mm) |
| **Screening for coronary artery disease in asymptomatic individuals** |  |  | **Patients With Prior CABG Surgery With Stable Chest Pain** |
| When coronary artery calcification findings are available from previous chest CT scans, using these findings to enhance risk stratification and guide treatment of modifiable risk factors should be considered. | IIa / C | 2a / C | In patients who have had prior CABG surgery presenting with stable chest pain who are suspected to have myocardial ischemia, it is reasonable to perform stress imaging or CCTA to evaluate for myocardial ischemia or graft stenosis or occlusion |
| Coronary artery calcium scoring (CACS) may be considered to improve risk classification around treatment decision thresholds. | IIb / C |  | **Patients With Known Non obstructive CAD Presenting With Stable Chest Pain** |
| **Symptomatic patients with established chronic coronary syndromes** |  | 2a / B | For symptomatic patients with known non obstructive CAD who have stable chest pain, CCTA is reasonable for determining atherosclerotic plaque burden and progression to obstructive CAD, and guiding therapeutic decision-making |
| In CCS patients with symptoms refractory to medical treatment, and who have had previous coronary revascularization, CCTA should be considered to evaluate bypass graft or stent patency (for stents ≥3 mm) | IIa / B |  | **Follow-up** |
| **Non-invasive functional myocardial imaging tests in the initial diagnostic management of individuals with suspected chronic coronary syndrome—resting and stress single-photon emission computed tomography/positron emission tomography—cardiac magnetic resonance imaging, if available and supported by local expertise** |  | 3 / B | In patients with CCD without a change in clinical or functional status on optimized GDMT, routine periodic testing with coronary CTA or stress testing with or without imaging is not recommended to guide therapeutic decision-making. |
| In patients selected for PET or SPECT myocardial perfusion imaging, it is recommended to measure CACS from unenhanced chest CT imaging (used for attenuation correction) to improve detection of both non-obstructive and obstructive CAD. | I / B |  |  |

**Supplemental Table 5**: ESC and ACC/AHA recommendations for cardiac rhythm disorders in 2024

| **ESC** | **COR / LOE** | | **ACC / AHA** |
| --- | --- | --- | --- |
| **Recommendations regarding imaging before implantation** |  |  | **Bradycardia and cardiac conduction delay** |
| Cardiac imaging is recommended in patients with suspected or documented symptomatic bradycardia to evaluate the presence of structural heart disease, to determine LV systolic function, and to diagnose potential causes of conduction disturbances. | I / C | 2a / C | In selected patients with bradycardia or bundle branch block, disease-specific advanced imaging (e.g., transesophageal echocardiography, computed tomography, cardiac magnetic resonance imaging [MRI], or nuclear imaging) is reasonable if structural heart disease is suspected yet not confirmed by other diagnostic modalities |
| Multimodality imaging (CMR, CT, or PET) should be considered for myocardial tissue characterization in the diagnosis of specific pathologies associated with conduction abnormalities needing pacemaker implantation, particularly in patients younger than 60 years | IIa / C | 2a / C | In selected patients with LBBB in whom structural heart disease is suspected and echocardiogram is unrevealing, advanced imaging (e.g., cardiac MRI, computed tomography, or nuclear studies) is reasonable |
| **Recommendations for anticoagulation in patients undergoing catheter ablation** |  |  | **Ventricular arrythmias** |
| Cardiac imaging (TOE or delayed phase computed tomography) should be considered prior to catheter ablation of AF in patients at high risk of ischemic stroke and thromboembolism despite taking oral anticoagulation to exclude thrombus | IIa / B | 1 / C | In patients presenting with VA who are suspected of having structural heart disease, cardiac magnetic resonance imaging (MRI) or computed tomography (CT) can be useful to detect and characterize underlying structural heart disease |
|  |  | 2a / C | In patients who have recovered from unexplained SCA, CT or invasive coronary angiography is useful to confirm the presence or absence of ischemic heart disease and guide decisions for myocardial revascularization. |
|  |  |  | **Syncope** |
|  |  | 2b / B | Computed tomography (CT) or magnetic resonance imaging (MRI) may be useful in selected patients presenting with syncope of suspected cardiac etiology |

**Supplemental Table 6**: ESC and ACC/AHA recommendations for congenital heart disease (CHD) in 2024

| **ESC** | **COR / LOE** | | **ACC / AHA** |
| --- | --- | --- | --- |
|  |  | 2a / C | CCT imaging can be use full in patients with ACHD when information that cannot be obtained by other diagnostic modalities is important enough to justify the exposure to ionizing radiation |
|  |  | 2a / B | In patients with a low or intermediate pretest probability of coronary artery disease (CAD), use of CT coronary angiography is reasonable to exclude significant obstructive CAD when cardiac catheterization has significant risk or because of patient preference |
|  |  | 1 / B | CMR, CCT, and/or TEE are useful to evaluate pulmonary venous connections in adults with atrial septal defect |
|  |  | 1 / B | CMR or CTA is recommended for evaluation of partial anomalous pulmonary venous connection |
|  |  | 1 / C | Aortic imaging using TTE, TEE, CMR, or CTA is recommended in adults with Williams syndrome or patients suspected of having supravalvular aortic stenosis |
|  |  | 1 / C | Coronary angiography, using catheterization, CT, or CMR, is recommended for evaluation of anomalous coronary artery |

**Supplemental Table 7**: ESC and ACC/AHA recommendations for cardiomyopathy and pericardial disease in 2024

| **ESC** | **COR / LOE** | | **ACC / AHA** |
| --- | --- | --- | --- |
| **Cardiomyopathy - Recommendations for computed tomography and nuclear imaging** |  |  | **Hypertrophic cardiomyopathy** |
| Contrast-enhanced cardiac CT should be considered in patients with suspected cardiomyopathy who have inadequate echocardiographic imaging and contraindications to CMR. | IIa / C | 2b / C | In adult patients with suspected HCM, cardiac CT maybe considered for diagnosis if the echocardiogram is not diagnostic and CMR imaging is unavailable |
| In patients with suspected cardiomyopathy, CT-based imaging should be considered to exclude congenital or acquired coronary artery disease as a cause of the observed myocardial abnormality | IIa / C | 1 / B | In patients with HCM with symptoms or evidence of myocardial ischemia, coronary angiography (CT or invasive) is recommended |
|  |  | 1 / B | ﻿In patients with HCM who are at risk of coronary atherosclerosis, coronary angiography (CT or invasive) is recommended before surgical myectomy |
| **Diagnostic workup of pericardial disease** |  |  | **Suspected myopericarditis** |
| CT and/or CMR are recommended as second-level testing for diagnostic workup in pericarditis | II / C | 2b / C | In patients with acute chest pain with suspected acute pericarditis, non-contrast or contrast cardiac CT scanning may be reasonable to determine the presence and degree of pericardial thickening |
| **Pericardial effusion** |  |  |  |
| CT or CMR should be considered in suspected cases of loculated pericardial effusion, pericardial thickening and masses, as well as associated chest abnormalities | IIa / C |  |  |
| Urgent imaging technique (transthoracic echocardiogram or CT) is indicated in patients with a history of chest trauma and systemic arterial hypotension | I / B |  |  |
| **Constrictive pericarditis** |  |  |  |
| CT and/or CMR are indicated as second-level imaging techniques to assess calcifications (CT), pericardial thickness, degree and extension of pericardial involvement | I / C |  |  |

**Supplemental Table 8**: ESC and ACC/AHA recommendations for VHD in 2024

| **ESC** | **COR / LOE** | | **ACC / AHA** |
| --- | --- | --- | --- |
| **Management of CAD in patients with VHD** |  |  | **Management of CAD in Patients Undergoing TAVI or Valve Surgery** |
| Coronary CT angiography should be considered as an alternative to coronary angiography before valve surgery in patients with severe VHD and low probability of CAD | IIa / C | 1 / C | In patients undergoing TAVI, 1) contrast-enhanced coronary CT angiography (in patients with a low pretest probability for CAD) or 2) an invasive coronary angiogram is recommended to assess coronary anatomy and guide revascularization. |
|  |  | 2a / B | In selected patients with a low to intermediate pretest probability of CAD, contrast-enhanced coronary CT angiography is reasonable to exclude the presence of significant obstructive CAD |
|  |  |  | **Initial Diagnosis of Aortic Stenosis** |
|  |  | 2a / B | In patients with suspected low-flow, low-gradient severe AS with normal or reduced LVEF (Stages D2 and D3), measurement of aortic valve calcium score by CT imaging is reasonable to further define severity |
|  |  |  | **Bicuspid Aortic Valve** |
|  |  | 1 / C | In patients with BAV, CMR angiography or CT angiography is indicated when morphology of the aortic sinuses, sinotubular junction, or ascending aorta cannot be assessed accurately or fully by echocardiography. |
|  |  | 2a / C | In patients with BAV and a diameter of the aortic sinuses or ascending aorta of >4.0 cm, lifelong serial evaluation of the size and morphology of the aortic sinuses and ascending aorta by echocardiography, CMR, or CT angiography is reasonable, with the examination interval determined by the degree and rate of progression of aortic dilation and by family history |
|  |  |  | **Chronic Secondary Mitral Regurgitation** |
|  |  | 1 / C | In patients with chronic secondary MR (Stages B to D), noninvasive imaging (stress nuclear/PET, CMR, or stress echocardiography), coronary CT angiography, or coronary arteriography is useful to establish etiology of MR and to assess myocardial viability. |
|  |  |  | **Diagnosis and Follow-Up of Prosthetic Valves** |
|  |  | 1 / C | In patients with a prosthetic valve replacement or prior valve repair and clinical symptoms or signs that suggest prosthetic valve dysfunction, additional imaging with TEE, gated cardiac CT, or fluoroscopy is recommended, even if TTE does not show valve dysfunction |
|  |  | 1 / C | In patients with mechanical valve stenosis, fluoroscopy or cine-CT is recommended to assess motion of the mechanical valve leaflets. |
|  |  | 1 / B | In patients with suspected mechanical prosthetic valve thrombosis, urgent evaluation with TTE, TEE, fluoroscopy, and/or multidetector CT imaging is indicated to assess valve function, leaflet motion, and the presence and extent of thrombus |
|  |  | 2a / C | In patients with suspected bioprosthetic valve thrombosis, 3D TEE or 4D CT imaging can be useful to rule-out leaflet thrombosis |

**Supplemental Table 9**: ESC and ACC/AHA recommendations for CV prevention in 2024

| **ESC** | **COR / LOE** | | **ACC / AHA** |
| --- | --- | --- | --- |
| **Cardiovascular imaging for risk assessment of atherosclerotic cardiovascular disease** |  |  | **Primary prevention and blood cholesterol** |
| CAC score assessment with CT may be considered as a risk modifier in the CV risk assessment of asymptomatic individuals at low or moderate risk | IIb / B | 2a / B | In intermediate-risk or selected borderline-risk adults, if the decision about statin use remains uncertain, it is reasonable to use a CAC score in the decision to withhold, postpone or initiate statin therapy |
| CAC scoring may be considered to improve risk classification around treatment decision thresholds. Plaque detection by carotid ultrasound is an alternative when CAC scoring is unavailable or not feasible | IIb / B | 2a / B | In intermediate-risk adults or selected borderline-risk adults in whom a CAC score is measured for the purpose of making a treatment decision, AND   - If the coronary calcium score is zero, it is reasonable to withhold statin therapy and reassess in 5 to 10 years, as long as higher risk conditions are absent (diabetes mellitus, family history of premature CHD, cigarette smoking). - If CAC score is 1 to 99, it is reasonable to initiate statin therapy for patients ≥ 55 years of age. - If CAC score is 100 or higher or in the 75th percentile or higher, it is reasonable to initiate statin therapy |
| After assessing 10-year predicted CVD risk and non-traditional CVD risk modifiers, if a risk-based BP-lowering treatment decision remains uncertain for individuals with elevated BP, measuring CAC score, carotid or femoral plaque using ultrasound, high-sensitivity cardiac troponin or B-type natriuretic peptide biomarkers, or arterial stiffness using pulse wave velocity, may be considered to improve risk stratification among patients with borderline increased 10-year CVD risk (5% to <10% risk) after shared decision-making and considering costs | IIb / B | 2b / B | In adults 76 to 80 years of age with an LDL-C level of 70 to 189 mg/dL (1.7 to 4.8 mmol/L), it may be reasonable to measure CAC to reclassify those with a CAC score of zero to avoid statin therapy |
|  |  | 2a / B | In adults at intermediate risk (≥ 7.5% to < 20% 10-year ASCVD risk) or selected adults at borderline risk (5% to < 7.5% 10-year ASCVD risk), if risk-based decisions for preventive interventions (e.g., statin therapy) remain uncertain, it is reasonable to measure a coronary artery calcium score to guide clinician–patient risk discussion |
|  |  | 2a / B | In intermediate-risk (≥ 7.5% to <20% 10-year ASCVD risk) adults or selected borderline-risk (5% to <7.5% 10-year ASCVD risk) adults in whom a coronary artery calcium score is measured for the purpose of making a treatment decision, AND   - If the coronary artery calcium score is zero, it is reasonable to withhold statin therapy and reassess in 5 to 10 years, as long as higher-risk conditions are absent (e.g., diabetes, family history of premature CHD, cigarette smoking) - If coronary artery calcium score is 1 to 99, it is reasonable to initiate statin therapy for patients ≥ 55 years of age - If coronary artery calcium score is 100 or higher or in the 75th percentile or higher, it is reasonable to initiate statin therapy |

**Supplemental Table 10**: ESC and ACC/AHA recommendations for sports cardiology in 2024

| **ESC** | **COR / LOE** | | **ACC / AHA** |
| --- | --- | --- | --- |
| **Recommendations for cardiovascular evaluation and regular exercise in healthy individuals aged >35 years** |  |  |  |
| In selected individuals without known CAD who have very high CVD risk (e.g. SCORE>10%, strong family history, or familial hypercholesterolemia) and want to engage in high- or very high-intensity exercise, risk assessment with a functional imaging test, coronary CCTA, or carotid or femoral artery ultrasound imaging may be considered. | IIb / B |  |  |
| **Recommendations for exercise in young individuals/athletes with anomalous origins of coronary arteries** |  |  |  |
| When considering sports activities, evaluation with imaging tests to identify high-risk patterns and an exercise stress test to check for ischemia should be considered in individuals with AOCA (anomalous origin of coronary arteries). | IIa / C |  |  |

**Supplemental Table 11**: ESC and ACC/AHA recommendations for endocarditis in 2024

| **ESC** | **COR / LOE** | | **ACC / AHA** |
| --- | --- | --- | --- |
| **Recommendations for the role of computed tomography, nuclear imaging, and magnetic resonance in infective endocarditis** |  |  | **Diagnosis of infective endocarditis** |
| Cardiac CTA is recommended in patients with possible NVE to detect valvular lesions and confirm the diagnosis of IE. | I / B | 2a / B | In patients in whom the anatomy cannot be clearly delineated by echocardiography in the setting of suspected paravalvular infections, CT imaging is reasonable |
| [18F] FDG-PET/CT(A) and cardiac CTA are recommended in possible PVE to detect valvular lesions and confirm the diagnosis of IE. | I / B |  |  |
| Cardiac CTA is recommended in NVE and PVE to diagnose paravalvular or periprosthetic complications if echocardiography is inconclusive. | I / B |  |  |
| **Recommendations for pre-operative coronary anatomy assessment in patients requiring surgery for infective endocarditis** |  |  |  |
| In hemodynamically stable patients with aortic valve vegetations who require cardiac surgery and are high risk of CAD, a high-resolution multislice coronary CTA is recommended. | I / B |  |  |

**Supplemental Table 12**: ESC and ACC/AHA recommendations cardio-oncology in 2024

| **ESC** | **COR / LOE** | | **ACC / AHA** |
| --- | --- | --- | --- |
| **Diagnosis and management of Takotsubo syndrome in patients with cancer** |  |  |  |
| Coronary angiography (invasive or CCTA) is recommended to exclude ACS. | I / C |  |  |
| **Recommendations for cardiovascular surveillance in asymptomatic adult cancer survivors** |  |  |  |
| Non-invasive screening for CAD (Stress echocardiography, cardiac CT, stress CMR, single-photon emission CT stress test, according to local protocol) should be considered every 5–10 years in asymptomatic patients who received 0.15 Gy MHD (mean heart dose) starting at 5 years after radiation. | IIa / C |  |  |
| **Diagnosis and management of ICI-associated pericarditis** |  |  |  |
| Multimodality CV imaging (echocardiography, CMR + CT), ECG and measurement of cardiac biomarkers are recommended to confirm the diagnosis, assess the hemodynamic consequences of pericardial disease, and rule out associated myocarditis | I / C |  |  |

**Supplemental Table 13**: ESC and ACC/AHA recommendations for heart failure in 2024

| **ESC** | **COR / LOE** | | **ACC / AHA** |
| --- | --- | --- | --- |
| CTCA should be considered in patients with a low to intermediate pre-test probability of CAD or those with equivocal non-invasive stress tests in order to rule out coronary artery stenosis | IIa / C | 1 / C | In patients for whom echocardiography is inadequate, alternative imaging (e.g., cardiac magnetic resonance [CMR], cardiac computed tomography [CT], radionuclide imaging) is recommended for assessment of LVEF |

#### **References**

1. McEvoy JW, McCarthy CP, Bruno RM, et al. 2024 ESC Guidelines for the management of elevated blood pressure and hypertension. *Eur Heart J*. 2024;45(38):3912-4018. doi:10.1093/eurheartj/ehae178

2. Bushnell C, Kernan WN, Sharrief AZ, et al. 2024 Guideline for the Primary Prevention of Stroke: A Guideline From the American Heart Association/American Stroke Association. *Stroke*. 2024;55(12). doi:10.1161/STR.0000000000000475

3. Vrints C, Andreotti F, Koskinas KC, et al. 2024 ESC Guidelines for the management of chronic coronary syndromes. *Eur Heart J*. 2024;45(36):3415-3537. doi:10.1093/eurheartj/ehae177

4. Thompson A, Fleischmann KE, Smilowitz NR, et al. 2024 AHA/ACC/ACS/ASNC/HRS/SCA/SCCT/SCMR/SVM Guideline for Perioperative Cardiovascular Management for Noncardiac Surgery: A Report of the American College of Cardiology/American Heart Association Joint Committee on Clinical Practice Guidelines. *Circulation*. 2024;150(19). doi:10.1161/CIR.0000000000001285

5. Joglar JA, Chung MK, Armbruster AL, et al. 2023 ACC/AHA/ACCP/HRS Guideline for the Diagnosis and Management of Atrial Fibrillation: A Report of the American College of Cardiology/American Heart Association Joint Committee on Clinical Practice Guidelines. *Circulation*. 2024;149(1). doi:10.1161/CIR.0000000000001193

6. Ommen SR, Ho CY, Asif IM, et al. 2024 AHA/ACC/AMSSM/HRS/PACES/SCMR Guideline for the Management of Hypertrophic Cardiomyopathy: A Report of the American Heart Association/American College of Cardiology Joint Committee on Clinical Practice Guidelines. *J Am Coll Cardiol*. Published online May 8, 2024. doi:10.1016/j.jacc.2024.02.014

7. Mazzolai L, Teixido-Tura G, Lanzi S, et al. 2024 ESC Guidelines for the management of peripheral arterial and aortic diseases. *Eur Heart J*. 2024;45(36):3538-3700. doi:10.1093/eurheartj/ehae179

8. McDonagh TA, Metra M, Adamo M, et al. 2023 Focused Update of the 2021 ESC Guidelines for the diagnosis and treatment of acute and chronic heart failure: Developed by the task force for the diagnosis and treatment of acute and chronic heart failure of the European Society of Cardiology (ESC) With the special contribution of the Heart Failure Association (HFA) of the ESC. *Eur Heart J*. 2023;44(37):3627-3639. doi:10.1093/eurheartj/ehad195

9. Virani SS, Newby LK, Arnold SV, et al. 2023 AHA/ACC/ACCP/ASPC/NLA/PCNA Guideline for the Management of Patients With Chronic Coronary Disease. *J Am Coll Cardiol*. 2023;82(9):833-955. doi:10.1016/j.jacc.2023.04.003

10. Byrne RA, Rossello X, Coughlan JJ, et al. 2023 ESC Guidelines for the management of acute coronary syndromes: Developed by the task force on the management of acute coronary syndromes of the European Society of Cardiology (ESC). *Eur Heart J*. 2023;44(38):3720-3826. doi:10.1093/eurheartj/ehad191

11. Facc EMI. 2022 ACC/AHA Guideline for the Diagnosis and Management of Aortic Disease. 2022;80(24).

12. Arbelo E, Protonotarios A, Gimeno JR, et al. 2023 ESC Guidelines for the management of cardiomyopathies: Developed by the task force on the management of cardiomyopathies of the European Society of Cardiology (ESC). *Eur Heart J*. 2023;44(37):3503-3626. doi:10.1093/eurheartj/ehad194

13. Heidenreich PA, Bozkurt B, Aguilar D, et al. 2022 AHA/ACC/HFSA Guideline for the Management of Heart Failure: Executive Summary. *J Am Coll Cardiol*. 2022;79(17):1757-1780. doi:10.1016/j.jacc.2021.12.011

14. Marx N, Federici M, Schütt K, et al. 2023 ESC Guidelines for the management of cardiovascular disease in patients with diabetes: Developed by the task force on the management of cardiovascular disease in patients with diabetes of the European Society of Cardiology (ESC). *Eur Heart J*. 2023;44(39):4043-4140. doi:10.1093/eurheartj/ehad192

15. Lawton JS, Tamis-Holland JE, Bangalore S, et al. 2021 ACC/AHA/SCAI Guideline for Coronary Artery Revascularization. *J Am Coll Cardiol*. 2022;79(2):e21-e129. doi:10.1016/j.jacc.2021.09.006

16. Delgado V, Ajmone Marsan N, de Waha S, et al. 2023 ESC Guidelines for the management of endocarditis: Developed by the task force on the management of endocarditis of the European Society of Cardiology (ESC) Endorsed by the European Association for Cardio-Thoracic Surgery (EACTS) and the European Association of Nuclear Medicine (EANM). *Eur Heart J*. 2023;44(39):3948-4042. doi:10.1093/eurheartj/ehad193

17. Gulati M, Levy PD, Mukherjee D, et al. 2021 AHA/ACC/ASE/CHEST/SAEM/SCCT/SCMR Guideline for the Evaluation and Diagnosis of Chest Pain. *J Am Coll Cardiol*. 2021;78(22):e187-e285. doi:10.1016/j.jacc.2021.07.053

18. Lyon AR, López-Fernández T, Couch LS, et al. 2022 ESC Guidelines on cardio-oncology developed in collaboration with the European Hematology Association (EHA), the European Society for Therapeutic Radiology and Oncology (ESTRO) and the International Cardio-Oncology Society (IC-OS). *Eur Heart J*. 2022;43(41):4229-4361. doi:10.1093/eurheartj/ehac244

19. Otto CM, Nishimura RA, Bonow RO, et al. 2020 ACC/AHA Guideline for the Management of Patients With Valvular Heart Disease. *J Am Coll Cardiol*. 2021;77(4):e25-e197. doi:10.1016/j.jacc.2020.11.018

20. Zeppenfeld K, Tfelt-Hansen J, De Riva M, et al. 2022 ESC Guidelines for the management of patients with ventricular arrhythmias and the prevention of sudden cardiac death. *Eur Heart J*. 2022;43(40):3997-4126. doi:10.1093/eurheartj/ehac262

21. Levine GN, O’Gara PT, Beckman JA, et al. Recent Innovations, Modifications, and Evolution of ACC/AHA Clinical Practice Guidelines: An Update for Our Constituencies: A Report of the American College of Cardiology/American Heart Association Task Force on Clinical Practice Guidelines. *Circulation*. 2019;139(17). doi:10.1161/CIR.0000000000000651

22. Halvorsen S, Mehilli J, Cassese S, et al. 2022 ESC Guidelines on cardiovascular assessment and management of patients undergoing non-cardiac surgery. *Eur Heart J*. 2022;43(39):3826-3924. doi:10.1093/eurheartj/ehac270

23. Arnett DK, Blumenthal RS, Albert MA, et al. 2019 ACC/AHA Guideline on the Primary Prevention of Cardiovascular Disease. *J Am Coll Cardiol*. 2019;74(10):e177-e232. doi:10.1016/j.jacc.2019.03.010

24. Humbert M, Kovacs G, Hoeper MM, et al. 2022 ESC/ERS Guidelines for the diagnosis and treatment of pulmonary hypertension. *Eur Heart J*. 2022;43(38):3618-3731. doi:10.1093/eurheartj/ehac237

25. Grundy SM, Stone NJ, Bailey AL, et al. 2018 AHA/ACC/AACVPR/AAPA/ABC/ACPM/ADA/AGS/APhA/ASPC/NLA/PCNA Guideline on the Management of Blood Cholesterol. *J Am Coll Cardiol*. 2019;73(24):e285-e350. doi:10.1016/j.jacc.2018.11.003

26. Visseren FLJ, Mach F, Smulders YM, et al. 2021 ESC Guidelines on cardiovascular disease prevention in clinical practice. *Eur Heart J*. 2021;42(34):3227-3337. doi:10.1093/eurheartj/ehab484

27. Kusumoto FM, Schoenfeld MH, Barrett C, et al. 2018 ACC/AHA/HRS Guideline on the Evaluation and Management of Patients With Bradycardia and Cardiac Conduction Delay. *J Am Coll Cardiol*. 2019;74(7):e51-e156. doi:10.1016/j.jacc.2018.10.044

28. Glikson M, Nielsen JC, Kronborg MB, et al. 2021 ESC Guidelines on cardiac pacing and cardiac resynchronization therapy. *Eur Heart J*. 2021;42(35):3427-3520. doi:10.1093/eurheartj/ehab364

29. Stout KK, Daniels CJ, Aboulhosn JA, et al. 2018 AHA/ACC Guideline for the Management of Adults With Congenital Heart Disease. *J Am Coll Cardiol*. 2019;73(12):e81-e192. doi:10.1016/j.jacc.2018.08.1029

30. Vahanian A, Beyersdorf F, Praz F, et al. 2021 ESC/EACTS Guidelines for the management of valvular heart disease: Developed by the Task Force for the management of valvular heart disease of the European Society of Cardiology (ESC) and the European Association for Cardio-Thoracic Surgery (EACTS). *Rev Esp Cardiol Engl Ed*. 2022;75(6):524. doi:10.1016/j.rec.2022.05.006

31. Whelton PK, Carey RM, Aronow WS, et al. 2017 ACC/AHA/AAPA/ABC/ACPM/AGS/APhA/ASH/ASPC/NMA/PCNA Guideline for the Prevention, Detection, Evaluation, and Management of High Blood Pressure in Adults. *J Am Coll Cardiol*. 2018;71(19):e127-e248. doi:10.1016/j.jacc.2017.11.006

32. McDonagh TA, Metra M, Adamo M, et al. 2021 ESC Guidelines for the diagnosis and treatment of acute and chronic heart failure. *Eur Heart J*. 2021;42(36):3599-3726. doi:10.1093/eurheartj/ehab368

33. Al-Khatib SM, Stevenson WG, Ackerman MJ, et al. 2017 AHA/ACC/HRS Guideline for Management of Patients With Ventricular Arrhythmias and the Prevention of Sudden Cardiac Death: Executive Summary. *J Am Coll Cardiol*. 2018;72(14):1677-1749. doi:10.1016/j.jacc.2017.10.053

34. Pelliccia A, Sharma S, Gati S, et al. 2020 ESC Guidelines on sports cardiology and exercise in patients with cardiovascular disease. *Eur Heart J*. 2021;42(1):17-96. doi:10.1093/eurheartj/ehaa605

35. Shen WK, Sheldon RS, Benditt DG, et al. 2017 ACC/AHA/HRS Guideline for the Evaluation and Management of Patients With Syncope: A Report of the American College of Cardiology/American Heart Association Task Force on Clinical Practice Guidelines and the Heart Rhythm Society. *Circulation*. 2017;136(5). doi:10.1161/CIR.0000000000000499

36. Baumgartner H, De Backer J, Babu-Narayan SV, et al. 2020 ESC Guidelines for the management of adult congenital heart disease. *Eur Heart J*. 2021;42(6):563-645. doi:10.1093/eurheartj/ehaa554

37. Chan WV, Pearson TA, Bennett GC, et al. ACC/AHA Special Report: Clinical Practice Guideline Implementation Strategies: A Summary of Systematic Reviews by the NHLBI Implementation Science Work Group: A Report of the American College of Cardiology/American Heart Association Task Force on Clinical Practice Guidelines. *Circulation*. 2017;135(9). doi:10.1161/CIR.0000000000000481

38. Brugada J, Katritsis DG, Arbelo E, et al. 2019 ESC Guidelines for the management of patients with supraventricular tachycardiaThe Task Force for the management of patients with supraventricular tachycardia of the European Society of Cardiology (ESC). *Eur Heart J*. 2020;41(5):655-720. doi:10.1093/eurheartj/ehz467

39. Levine GN, Bates ER, Bittl JA, et al. 2016 ACC/AHA Guideline Focused Update on Duration of Dual Antiplatelet Therapy in Patients With Coronary Artery Disease: A Report of the American College of Cardiology/American Heart Association Task Force on Clinical Practice Guidelines: An Update of the 2011 ACCF/AHA/SCAI Guideline for Percutaneous Coronary Intervention, 2011 ACCF/AHA Guideline for Coronary Artery Bypass Graft Surgery, 2012 ACC/AHA/ACP/AATS/PCNA/SCAI/STS Guideline for the Diagnosis and Management of Patients With Stable Ischemic Heart Disease, 2013 ACCF/AHA Guideline for the Management of ST-Elevation Myocardial Infarction, 2014 AHA/ACC Guideline for the Management of Patients With Non–ST-Elevation Acute Coronary Syndromes, and 2014 ACC/AHA Guideline on Perioperative Cardiovascular Evaluation and Management of Patients Undergoing Noncardiac Surgery. *Circulation*. 2016;134(10). doi:10.1161/CIR.0000000000000404

40. Mach F, Baigent C, Catapano AL, et al. 2019 ESC/EAS Guidelines for the management of dyslipidaemias: lipid modification to reduce cardiovascular risk. *Eur Heart J*. 2020;41(1):111-188. doi:10.1093/eurheartj/ehz455

41. Halperin JL, Levine GN, Al-Khatib SM, et al. Further Evolution of the ACC/AHA Clinical Practice Guideline Recommendation Classification System.

42. Konstantinides SV, Meyer G, Becattini C, et al. 2019 ESC Guidelines for the diagnosis and management of acute pulmonary embolism developed in collaboration with the European Respiratory Society (ERS). *Eur Heart J*. 2020;41(4):543-603. doi:10.1093/eurheartj/ehz405

43. Page RL. 2015 ACC/AHA/HRS Guideline for the Management of Adult Patients With Supraventricular Tachycardia.

44. Neumann FJ, Sousa-Uva M, Ahlsson A, et al. 2018 ESC/EACTS Guidelines on myocardial revascularization. *Eur Heart J*. 2019;40(2):87-165. doi:10.1093/eurheartj/ehy394

45. 2014 AHA/ACC Guideline for the Management of Patients With Non–ST-Elevation Acute Coronary Syndromes.

46. Regitz-Zagrosek V, Roos-Hesselink JW, Bauersachs J, et al. 2018 ESC Guidelines for the management of cardiovascular diseases during pregnancy. *Eur Heart J*. 2018;39(34):3165-3241. doi:10.1093/eurheartj/ehy340

47. Arnett DK, Goodman RA, Halperin JL, Anderson JL, Parekh AK, Zoghbi WA. AHA/ACC/HHS Strategies to Enhance Application of Clinical Practice Guidelines in Patients With Cardiovascular Disease and Comorbid Conditions.

48. Brignole M, Moya A, De Lange FJ, et al. 2018 ESC Guidelines for the diagnosis and management of syncope. *Eur Heart J*. 2018;39(21):1883-1948. doi:10.1093/eurheartj/ehy037

49. Jacobs AK, Anderson JL, Halperin JL. The Evolution and Future of ACC/AHA Clinical Practice Guidelines: A 30-Year Journey. *J Am Coll Cardiol*. 2014;64(13):1373-1384. doi:10.1016/j.jacc.2014.06.001

50. Bueno H, Byrne RA, Collet JP, et al. 2017 ESC focused update on dual antiplatelet therapy in coronary artery disease developed in collaboration with EACTS.

51. O’Gara PT, Kushner FG, Ascheim DD, et al. 2013 ACCF/AHA Guideline for the Management of ST-Elevation Myocardial Infarction. *J Am Coll Cardiol*. 2013;61(4):e78-e140. doi:10.1016/j.jacc.2012.11.019

52. Adler Y, Charron P, Imazio M, et al. 2015 ESC Guidelines for the diagnosis and management of pericardial diseases. *Eur Heart J*. 2015;36(42):2921-2964. doi:10.1093/eurheartj/ehv318

53. Anderson L, Pennell D. The role of endomyocardial biopsy in the management of cardiovascular disease: a Scientific Statement from the American Heart Association, the American College of Cardiology, and the European Society of Cardiology. *Eur Heart J*. 2008;29(13):1696-1696. doi:10.1093/eurheartj/ehn189
